# Amine Modification of Silica Aerogels/Xerogels for Removal of Relevant Environmental Pollutants

**DOI:** 10.3390/molecules24203701

**Published:** 2019-10-15

**Authors:** Alyne Lamy-Mendes, Rafael B. Torres, João P. Vareda, David Lopes, Marco Ferreira, Vanessa Valente, Ana V. Girão, Artur J. M. Valente, Luísa Durães

**Affiliations:** 1 CIEPQPF, Department of Chemical Engineering, University of Coimbra, 3030-790 Coimbra, Portugal; alyne@eq.uc.pt (A.L.-M.); rtorres@eq.uc.pt (R.B.T.); jvareda@eq.uc.pt (J.P.V.); davidPned10@hotmail.com (D.L.); ferreiramarcojl@hotmail.com (M.F.); vanessa.valente1994@gmail.com (V.V.); 2 CICECO—Aveiro Institute of Materials, Department of Materials and Ceramic Engineering, University of Aveiro, 3810-193 Aveiro, Portugal; avgirao@ua.pt; 3 CQC, Department of Chemistry, University of Coimbra, 3004-535 Coimbra, Portugal; avalente@ci.uc.pt

**Keywords:** silica aerogels, amine modification, volatile organic compounds, heavy metals, textile dyes, adsorption

## Abstract

Serious environmental and health problems arise from the everyday release of industrial wastewater effluents. A wide range of pollutants, such as volatile organic compounds, heavy metals or textile dyes, may be efficiently removed by silica materials advanced solutions such as aerogels. This option is related to their exceptional characteristics that favors the adsorption of different contaminants. The aerogels performance can be selectively tuned by an appropriate chemical or physical modification of the aerogel’s surface. Therefore, the introduction of amine groups enhances the affinity between different organic and inorganic contaminants and the silica aerogels. In this work, different case studies are reported to investigate and better understand the role of these functional groups in the adsorption process, since the properties of the synthesized aerogels were significantly affected, regarding their microstructure and surface area. In general, an improvement of the removal efficiency after functionalization of aerogels with amine groups was found, with removal efficiencies higher than 90% for lead and Rubi Levafix CA. To explain the adsorption mechanism, both Langmuir and Freundlich models were applied; chemisorption is most likely the sorption type taking place in the studied cases.

## 1. Introduction

The growth of industrial complexes has led to a considerable contamination increase in aquatic environments, as a significant amount of different pollutants, both organic and inorganic, are released with wastewaters, reaching natural aquifers [1,2,3,4]. For example, substantial quantities of aromatic compounds are employed as industrial solvents and are present in petroleum and gasoline [5,6]. Heavy metals are released from mining, smelting and other metallurgic industries in great amounts; however, agriculture, transportation and energy production also significantly contribute to the increase of these pollutants [4,7,8]. Additionally, chemical dyes are employed in different industrial applications, namely in textiles, foods, cosmetics, pharmaceuticals or pesticides [9,10].

Due to their toxicity, associated with carcinogenic and mutagenic effects in some cases, there is a growing interest and need to remove these pollutants from wastewater effluents, from both health and environmental perspectives [3,10,11,12,13,14]. Nevertheless, this process is a recurrent challenge regarding volatile organic compounds (VOCs) due to their highly volatile nature and persistency in the environment [15]. Phenol, for example, is highly toxic even at low concentrations [16] and the exposure to pollutants such as benzene and xylene for long periods of time leads to several negative effects on human health, from skin irritation to cancer and liver lesions [17]. Regarding heavy metals, their non-degradability leads to their perseverance in the environment, while their bioavailability allows easy incorporation in the food chain. As such, they can be found in water, soil, air and in the tissues of living organisms, since bioaccumulation occurs [18,19]. On the other hand, dyes can cause the coloring of aquatic environments, which make their removal essential, since deep colors can hinder light penetration through water [20,21], affecting the reactions of photosynthesis by underwater plants and, consequently, influencing the production of oxygen in water and the viability of aquatic life [21]. Moreover, several dyes are highly stable in aqueous media, poorly biodegradable, light-resistant and toxic for humans, aquatic animals, plants and microorganisms [11,22].

For the aforementioned reasons, the more conventional wastewater treatments need to be improved, due to increasing difficulty to comply with regulations [23,24]. Among the different treatment technologies available as, for example, biological treatment, chemical oxidation, coagulation, precipitation and membrane separation, the adsorption process has aroused special interest [25,26]. Its effectiveness, easy adaptation, simple operation and the availability of different adsorbents make adsorption a viable method for a wide range of applications [19,24,27]. For the removal of pollutants from aqueous solutions, materials such as activated carbon, mesoporous SiO_2_, alumina, zeolites or clays are commonly applied [19,28,29,30]. However, the use of these predominant industrial sorbents is limited due to some drawbacks, including poor adsorption capacities, low removal efficiency and slow kinetics [19,31].

Silica aerogels are a good alternative to overcome these limitations via manipulation of sol-gel technology. A combination of high porosity, small pores and a versatile surface chemistry results in capable adsorbents [19,31,32,33]. In fact, a higher surface area favors adsorption performance and the latter can be further enhanced with surface functional groups or charge [34].

By changing the aerogels’ degree of hydrophobicity, Štandeker et al. [35], through the incorporation of methyltrimethoxysilane (MTMS) or trimethylethoxysilane (TMES) in tetramethyl orthosilicate (TMOS) based aerogels, were able to improve the removal of toxic organic compounds from water, such as benzene, toluene, chloroform and chlorobenzene. The synthesized superhydrophobic materials showed adsorption capacities 15 to 400 times higher than the values obtained by granulated active carbon. Qin et al. [36] changed the hydrophilic character of tetraethyl orthosilicate (TEOS) based aerogels by modifying these materials with trimethylchlorosilane (TMCS). With these aerogels, it was possible to achieve high removal rates of phenol from water, with a maximum adsorption capacity of 142 mg/g when the equilibrium concentration of phenol was 290 mg/L.

For the adsorption of heavy metals, Štandeker [37] and Pouretedal [38] synthesized thiol modified silica aerogels using 3-mercaptopropyltrimethoxysilane (MPTMS) and TMOS or sodium silicate. Faghihian [39,40] and Ali [41] modified silica aerogels with amine groups using 3-aminopropyltrimethoxysilane (APTMS) or 3-aminopropyltriethoxysilane (APTES) and TEOS. Xiaonan et al. [42] prepared amine modified silica cryogels using [3-(trimethoxysilyl)propyl]-ethylenediamine (TMSEN) as the sole precursor to obtain an amine-bridged gel. Furthermore, our research group also modified silica aerogels with thiol and amine groups [43,44] for this purpose, using APTMS, MPTMS, methyltriethoxysilane (MTES) and TEOS. These materials feature enhanced adsorption properties when compared to activated carbon, biochar or natural zeolites [24], achieving adsorption capacities one order of magnitude greater in the best cases.

Han et al. [33] prepared both hydrophobic and hydrophilic silica aerogels derived from TEOS for the adsorption of methylene blue (MB) and rhodamine B (RhB). When modified with hexamethyldisilazane (HMDZ), the prepared aerogels showed strong affinity towards MB due to the dye’s better interaction with the attached methyl groups, achieving adsorption capacities of circa 65.7 mg/g. On the other hand, a hydrophilic material was preferred for the adsorption of RhB, as the carboxyl group of this pollutant can form hydrogen bonds with the hydroxyl groups of the synthesized aerogels, allowing an adsorption capacity of 185.6 mg/g. Wei et al. [45] fabricated hydrophilic silica aerogels based on TEOS for selective adsorption of cationic (MB, RhB and crystal violet) and anionic dyes (acid orange 7). The developed material showed specific surface area of 888.7 m^2^·g^−1^ and pore size from 2 to 55 nm, leading to a removal rate up to 90% for the cationic ones.

From the examples reported above, the interaction between silica aerogels and the pollutants can be improved by modifying the aerogels’ surface chemistry. Apart from the presence of methyl groups, the presence of amino and carboxyl functional groups can also result in strong affinities towards specific contaminants [10,19,43,46]. However, some of these modifications result in the decrease of the specific surface area [10,44]. In fact, Aguado et al. [47] showed that the incorporation of active amino functional groups resulted in low values of surface area down to 48 m^2^/g. Nevertheless, removal rates greater than 50% were obtained for a concentration of 100 mg/L of Cu, Zn, Cd and Pb. In fact, amine and carboxylic groups act as Lewis bases while heavy metal cations act as Lewis acids. The interactions between the two phases can be explained by the Pearson’s Hard and Soft Acids and Bases theory [48]. The same behavior of surface area decreasing was observed by Ho et al. [49] in mesoporous silica grafted with amino groups, used to remove acid blue 25 and MB dyes from wastewater. The authors reported maximum adsorption capacities of 256 and 90 mg/g for these dyes, respectively. Even though specific surface area is a key factor in the pollutants removal, several factors can be adjusted to enhance the overall adsorption rate, such as pH, contact time and contaminant concentration [45,50].

Due to the enhanced affinity/interaction of amino groups with several pollutants, the aim of this work is to show the influence of this functionalization on silica aerogels removal capacities for organic and inorganic contaminants in aqueous solutions. The chosen pollutants have high environmental relevance, owing to their toxicity and persistency, and are also commonly found in different industrial wastewaters. The obtained results are compared against other adsorbents reported in the literature for these pollutants.

## 2. Results and Discussion

### 2.1. Aerogels Selection Based on Preliminary Adsorption Tests

The compositions of aerogels and xerogels prepared in this work are presented in Table 1, which are based on the optimization of synthesis conditions performed in earlier works [51,52,53]. They have a common amine modification with the co-precursor APTMS, as reported in Section 3.

The drying methodology can have a significant influence on the final properties of silica materials, as a higher degree of shrinkage is usually observed in samples dried at ambient pressure, if compared with the ones dried under supercritical conditions. This shrinkage can be explained by the high affinity of the alcoholic solvent with the relevant amount of hydrophilic moieties in the silica structure of gels. The exceptions were the materials synthesized for the VOCs adsorption which were only dried at ambient pressure, as M and MA materials showed negligible shrinkages and properties very similar to aerogels as reported in a previous work [54]. Thus, preliminary adsorption tests were performed for ambient pressure and supercritically dried adsorbents, i.e., xerogels and aerogels, respectively, in order to reduce the number of materials in further adsorption tests. Table 1 shows the adsorption capacity of the adsorbents, at the equilibrium, when the highest initial concentration of the pollutants was tested.

Regarding the drying method, and because of significant changes on the microstructure of the materials, major differences are verified on the adsorption capacities of these pollutants. In fact, when the materials were applied in the removal of heavy metals, for most of the cases, the adsorption capacity seems to increase slightly in xerogels, compared with the aerogels’ counterparts. Therefore, the former were chosen to be applied for the adsorption tests of heavy metals, taking also into consideration the simplicity of the drying method. For the Rubi Levafix removal, a significant improvement was achieved when the aerogels were used as the adsorbents while, in the case of MB, both materials showed very similar adsorption capacities. For these reasons, the aerogels were the chosen materials for the removal of dyes.

Moreover, a significant effect of the amine modification was verified in the adsorption performance as, in most of the cases, amine modified materials showed higher values of pollutants uptake. Thus, in order to better understand the influence of this modification, the materials chosen to be applied in the adsorption tests were further characterized.

### 2.2. Properties of the Adsorbents

The physical and structural properties of the different silica-based xerogel and aerogel adsorbents developed in this work (with or without amine functionalization, for comparison purposes) are reported in Table 2.

M and MA materials, as already mentioned, showed properties similar to aerogels such as porosities superior than 94% and densities as low as 75.3 kg/m^3^ [54], as seen in Table 2. When amine groups were added to the MTMS system, a small increase in the density was verified, which can be justified by the higher degree of condensation of these materials [54]. For both materials, the obtained skeletal densities are in agreement with the ones previously reported in the literature for these materials [55,56]. The specific surface areas were evaluated from the adsorption branch of the isotherms applying the Brunauer–Emmett–Teller (BET) method, and the representative isotherms of the materials are shown in Figure S1. The modification of MTMS materials with amine groups caused a significant reduction in the surface area, explained by the large secondary silica particles radii and high amount of macropores (Table 2), as earlier reported by the authors [54]. In fact, the steric hindrance of propylamine groups along with their catalytic effect on condensation leads to a coarsening of the pearl necklace structure of these ambient dried aerogels. Finally, both MTMS-based aerogels showed super-hydrophobic properties, with contact angles above 160°, due to a high predominance of MTMS precursor. This non-wetting behavior can be confirmed by Fourier-Transform Infrared Spectroscopy (FTIR) analysis (Figure S2), and the significance of methyl groups is also proved by the appearance of the C-H bands between 2800 and 3000 cm^−1^ and near 1260 cm^−1^, along with a Si-C bond around 840 cm^−1^ [57]. Moreover, the typical Si-O-Si and O-Si-O bands were observed in the spectra, with these also appearing in the FTIR spectra of all the samples (Figure S2).

For the Mt materials, the amine addition caused an increase in the bulk density and a decrease in the porosity (Table 2), which is mainly related to the increase of hydrophilicity of the gel network, causing higher shrinkages during the drying step. Therefore, the modification with amine groups leads to a considerable reduction in the surface areas of Mt materials and a small increase in the average pore diameter, confirming the tendency verified for the MTMS-based materials. The presence of amine groups also changed the character of the samples towards to higher hydrophilicity.

In the case of A_T and A_TA, the low values of bulk densities and high porosities are mainly influenced by the drying method applied, indicating its relevance on the preservation of the microstructure of the gels even with hydrophilic skeletons. For the skeletal densities, the values are in agreement with the literature; for TMOS materials, values between 1700 and 1900 kg/m^3^ are expected [58], and for the samples modified with APTMS, a decrease can be expected [56]. Moreover, this introduction of amine groups on the TMOS-derived silica matrix caused a reduction in the surface area, along with an increase in the average pore diameter (Table 2), confirming the trend verified for the other systems here presented. Regarding the hydrophilic character, since both samples are obtained from TMOS, they feature a surface covered with hydroxyl groups, which was confirmed by the FTIR analysis (Figure S2); thus, they exhibit a significant affinity with water.

The bands associated with the amine containing precursor are not very clear in the FTIR spectra, as the characteristic bands are overlapped by more intense ones. However, there are slight changes in the spectra at 1600 cm^−1^ and 2850 cm^−1^, due to N-H bonds and CH_2_ groups, respectively, when comparing non-amine and amine-modified materials.

Elemental analysis was also performed to assess the mass fraction of specific chemical elements, namely C, H and N. This technique can provide information about the incorporation of amine groups in the adsorbents and about the extent of the sol-gel reactions (incomplete or complete condensation) [43]. The theoretical weight percentages of Si + O, C, H and N were calculated on the assumptions that: (1) all hydrolysable groups, from all the silica precursors, reacted—complete condensation; (2) one hydroxyl group per hydrolyzed precursor did not react—incomplete condensation 1OH and (3) two hydroxyl groups per hydrolyzed precursor did not react—incomplete condensation 2OH. For the theoretical data, it is also considered a complete hydrolysis of the precursors and that all precursors molecules condensed. The experimental values from elemental analysis and theoretical results related to these elements are shown in Table 3.

For the adsorbents developed without amine precursors, the experimental data are closer to the theoretical scenario of the complete condensation, while in the presence of amine groups, the experimental data indicate that an incomplete condensation occurs (Table 3). That incomplete condensation can be justified by the steric hindrance caused by the aminopropyl group, preventing the formation of siloxane bridges. For some samples, especially for the A_T adsorbent, higher values of C were observed, indicating that not all precursors underwent complete hydrolysis. In the case of VOCs and dyes’ adsorbents this discrepancy was expected, since for these materials the hydrolysis step is accomplished in very short periods. The presence of traces of N on the samples without the amine precursor is due to residues of ammonia catalyst [43]. The increase of N element for the samples synthesized with APTMS indicates the incorporation of this precursor in the silica network, proving a successful modification of the matrix. In addition, the nitrogen content is proportional to the molar ratio of APTMS in the precursor system, as expected.

**Figure 1:**
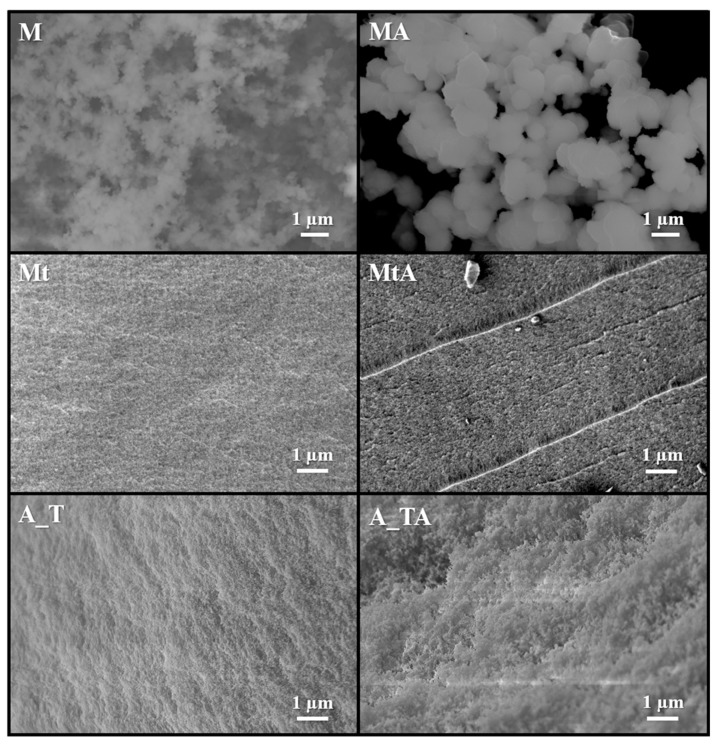
SEM images of the adsorbents M and MA (Reprinted with permission from Reference [54] Copyright (2019) Elsevier), and Mt, MtA, A_T and A_TA.

The microstructure of the different adsorbents is shown in Figure 1, with a magnification of 10,000×. In these images we observe that the morphology of the surface shows an interconnected silica network for all the samples. It is clear that the amine addition leads to a more open structure in all systems, with higher pore diameters, as also confirmed by the data presented in Table 2. The samples obtained with MTMS showed a much more open structure, with larger radii of secondary particles (especially in the material containing amine groups) when compared to the other systems. When TEOS or TMOS were used in the synthesis as network main builders, a more closed and smoother surface is observed, attributed to a higher degree of condensation of the silanol groups and uniformity of bonding in orthosilicates.

### 2.3. Adsorption of Pollutants

#### 2.3.1. Adsorption Equilibrium

**Figure 2:**
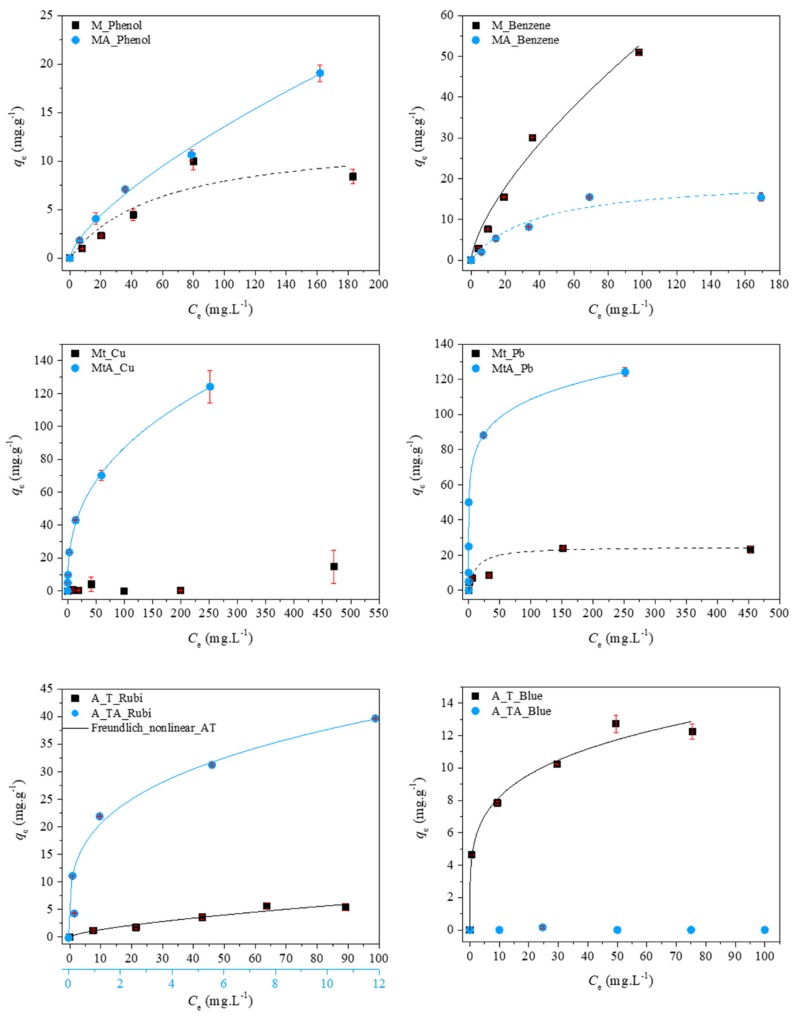
Experimental equilibrium data and the best fitted isotherm model for adsorption of the pollutants into the studied aerogels and xerogels. (dashed line—Langmuir; solid line—Freundlich).

The parameters of the Langmuir and Freundlich isotherm models are presented in Table 4 for all adsorbent-adsorbate pairs tested. Experimental equilibrium data, as well as the best isotherm model, are plotted in Figure 2.

The analysis of Table 4 and Figure 2 leads to two general conclusions: the prepared aerogel/xerogel materials remove all types of pollutants tested via adsorption and the modification with amine groups is, usually, significant in their adsorption performance.

Table 5 presents the removal efficiencies achieved by the adsorbents for three different initial concentrations (*C_i_*) of adsorbate.

**Scheme 1:**
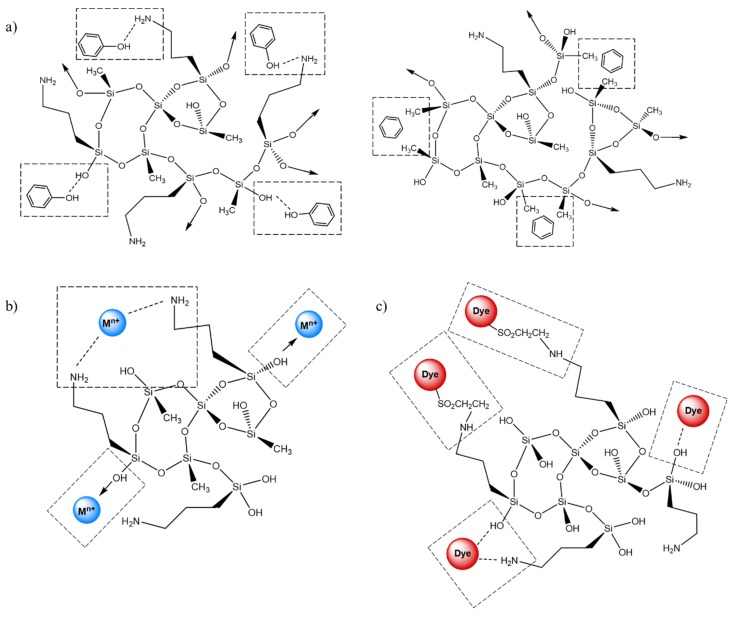
Proposed adsorption mechanisms between silica aerogels and (**a**) volatile organic compounds, (**b**) heavy metals and (**c**) textile dyes.

The adsorption of VOCs is achieved by both tested adsorbents, but the effect of the amine group addition is different depending on the adsorbate (Table 4 and Table 5). For phenol adsorption, the Langmuir model explains slightly better the interactions with the M adsorbent (although with an AIC value very close to the Freundlich model), while the Freundlich model better fitted the data for the MA adsorbent (Table 4). According to the values of the parameter (1/*n*_F_), the adsorption of this pollutant by the MA adsorbent is favorable, which is in agreement with the findings of Matias et al. [59], and the sorption mechanism is mostly affected by chemisorption [19]. A better phenol removal efficiency is observed with the MA adsorbent when compared to M, especially for an initial concentration of 200 mg/L, as seen in Table 5. This can be justified by the interaction of the amine group with the hydroxyl group of phenol molecules by hydrogen bonding, as represented in Scheme 1a. It is clear that the addition of amine groups improves the *q*_e_ when compared with pure MTMS-based material. The obtained values are higher than those reported for granular activated carbon (1.48 mg/g) [60], porous hydroxyapatite (8.2 mg/g) [61], calcinated clay (2.9 mg/g) [62] and MTMS xerogels (4.9 mg/g) [63], and are similar to *q*_e_ values achieved by MTMS aerogels (21.1 mg/g) [63] and modified activated carbon (20 mg/g) [64]. However, the developed materials showed lower adsorption capacities if compared with, for example, graphene aerogels–mesoporous silica frameworks, that have much higher surface area (1000.80 m^2^/g) [65]. The removal efficiencies of MA were also superior than those obtained by Matias et al. [59], using MTMS-based systems modified with Glymo and β-cyclodextrin for phenol adsorption.

For benzene, on the other hand, due to its non-polar nature, a higher removal is achieved with the M adsorbent (Table 5). For this pollutant, the Langmuir model explains the interactions with the MA adsorbent, while the Freundlich model better describes the interaction with the M adsorbent (Table 4). The heterogeneity factor obtained for the latter indicates that the removal of this pollutant is favorable. Hydrophobic interaction between the methyl groups derived from MTMS and the benzene molecule is the most likely adsorption mechanism in this system (Scheme 1a). For the latter, it cannot be ruled out that the poor fitting of Langmuir equation might be due to the fact that *q_e_* was not experimentally reached. As reported by Perdigoto et al. [63] the preparation method of the adsorbent materials can highly influence their adsorption capacities, as different synthesis procedures lead to different pore structures. Comparing the benzene adsorption capacity of MTMS material here developed with the ones obtained by Perdigoto et al. [63] and Štandeker et al. [35], this influence is clear. The lower adsorption capacities among the three works were obtained by Štandeker et al. [35], whose material presented low values of surface area combined with an average pore size of 4.8 nm. The ambient pressure dried (APD) aerogel developed in this work has similar surface area value to that obtained by Perdigoto et al. [63]. Nevertheless, the adsorption capacities herein reported were inferior to the ones reported there, which can be attributed to the different pore structures, as our average pore size is of 110.5 nm and the reported material of Ref. [63] has a bimodal distribution of micropores and mesopores. Thus, for benzene adsorption, the presence of both micropores and mesopores seems to be more significant to achieve higher removal rates in MTMS-based materials than the modification with amine groups.

In the case of heavy metal cations removal, the two prepared absorbents generated contrasting results: the Mt material removes very small amounts of pollutant (when used with copper, the adsorption follows a random pattern), while MtA adsorbent has very high affinity towards the cations. The presence of the amine groups in the prepared aerogel-like adsorbent is of upmost importance for the removal of heavy metals, always achieving removal efficiencies higher than 70%, even at high pollutant concentrations (Table 5). The highest amount of adsorbed copper and lead on adsorbent MtA is, curiously, the same in mass (Table 4). However, the adsorbent does not interact similarly with both cations. In fact, assuming that the most relevant interactions occurs between the metal ions and the amine non-bonding electrons (Scheme 1b), it comes out that the charge density of Pb(II) is much lower than that of Cu(II) as a consequence of their ionic radii (1.20 and 0.62 Å for Pb(H_2_O)_6_^2+^ and Cu(H_2_O)_4_^2+^, respectively) [66]. The adsorption capacity obtained with the xerogels under study is superior to that obtained with other amine modified silica aerogels, reported by Faghihian and co-authors (47.6 mg/g for copper, 45.5 mg/g for lead) [39,40]. The amine modified cryogels presented by Xiaonan [42] have a lower removal for copper (77 mg/g) than the xerogels of this work and a higher removal capacity for lead (276 mg/g). In the aforementioned studies, the adsorbents had greater surface areas (> 240 m^2^/g) than MtA adsorbent. The results shown in Table 4 and Table 5 are similar to the ones obtained previously by the authors [43,44] using amine and thiol groups in the same adsorbent (125 mg/g for copper and 139.9 mg/g for lead). This fact indicates that most of the removal ought to be due to the amine groups. Similar modifications to silica materials, such as chitosan (90.9 mg/g for lead) and thiol (37.8 mg/g for copper and 110.7 mg/g for lead), are not as efficient in removing copper and lead as the primary amine groups are [67,68]. For the adsorption of copper, the Freundlich isotherm fitted the data better than the Langmuir model. On the other hand, the Langmuir model fits the lead adsorption more accurately in the absence of amine groups, while for MtA, Freundlich is the more appropriate model (Table 4). Both models (considered the obtained parameters—Table 4) suggest that chemisorption is the type of adsorption mechanism taking place for pollutants. The Freundlich model reveals that the MtA adsorbent is fairly heterogenous and that the interactions toward lead are stronger, indicated by *K*_F_ values.

The addition of amine groups to the silica matrix expressively improved the interaction between the adsorbent and the Rubi dye, increasing the removal efficiency to above 85%, even at high values of initial concentration (Table 5). In this case, the Freundlich model was the one that better fitted the data (Table 4). This model indicates a favorable adsorption involving heterogeneous surfaces, as observed in Figure 2. The obtained heterogeneity factor indicates that the removal of the dye is due to chemisorption [19]. The Rubi dye, being part of a class of reactive dyes, comprises two principal functional groups, namely vinylsulfone and fluorotriazine [69,70], and, even though the chemical structure is not yet disclosed, the FTIR analysis of this dye confirmed the presence of these reactive groups (Figure S3). A possible mechanism may involve the interactions between the dye’s vinylsulfone groups (SO_2_CH=CH_2_) and the amino groups from the silica network (Scheme 1c). Such interactions were already reported for the adsorption of another reactive dye [71]. The OH and NH_2_ groups from the silica network can also form strong interactions, such as hydrogen bonds, with the groups from the dye. When compared with the A_T, that only has hydroxyl groups, the A_TA material shows a better removal efficiency, which can be justified by a synergistic effect between amine and hydroxyl groups in the removal of the Rubi Levafix CA. In addition, electrostatic (ion-ion or ion-dipole) interactions can occur between the Rubi Levafix CA and the adsorbent, i.e., the anionic character of the dye favors the interactions with the surface of the A_TA sample, that presents a zeta potential of +32.2 mV. Rubi is also more likely to be adsorbed by A_TA because reactive dyes usually are large molecules [69,72] and this adsorbent shows a more open network (Figure 1 and Table 2).

For MB, better removal efficiencies were achieved when A_T was used as the adsorbent (Table 5), with the Freundlich model better adjusting the experimental data (Table 4). Similar to Rubi removal with A_TA, the heterogeneity factor here obtained reveals a mechanism of adsorption driven by chemisorption. The MB small size (1.5 nm) [73] favors its adsorption even in materials with only micro and mesopores. In this case, the interaction between the blue dye and the adsorbent can be mainly attributed to electrostatic interactions, as the cationic character of MB favors its contact with the surface of the A_T sample, terminated with silanol groups, i.e., a negatively charged surface. These assumptions are in agreement with its negative zeta potential (−14.6 mV).

For the remaining comparative cases, the adsorbent A_T showed very low removal efficiencies in the case of Rubi, while the A_TA displayed negligible removal efficiencies for the MB (Table 5), indicating that these materials are not suitable for wastewaters treatment containing these dyes. These findings can be justified by the repulsion forces between the adsorbent and the pollutant with the same charges.

#### 2.3.2. Adsorption Kinetics

**Figure 3:**
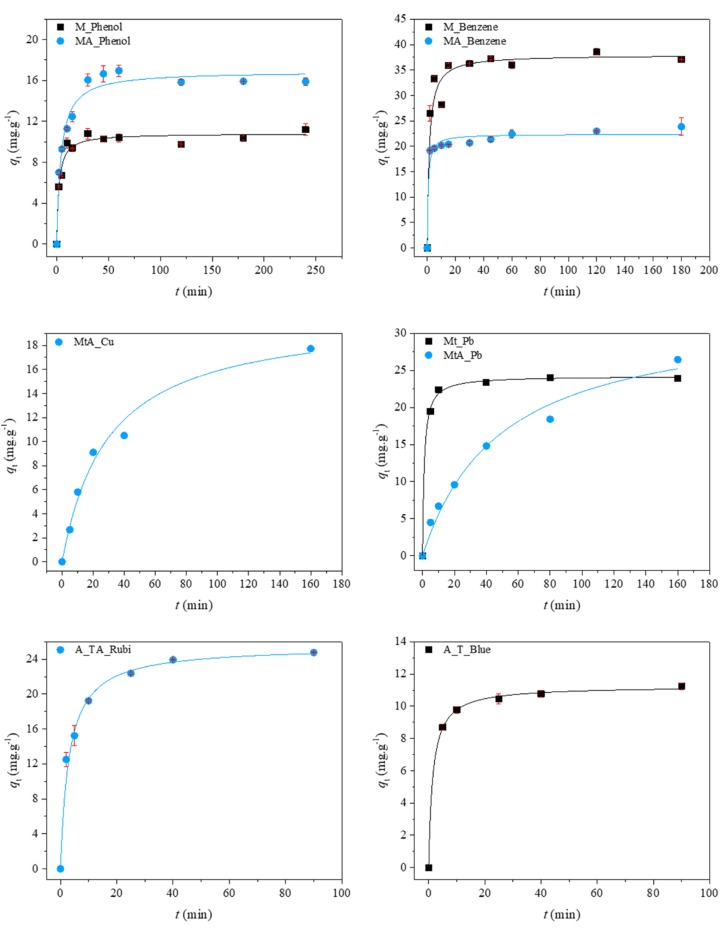
Representative plots of the fit of non-linearized form of pseudo-second order equation (solid line) to the data of experimental adsorption capacity as function of time, for the studied adsorbents towards VOCs (*C*_0_ = 100 ppm, 15 rpm, 20 °C), heavy metals (*C*_0_ = 200 ppm, 15 rpm, 20 °C) and dyes (*C*_0_ = 50 ppm, 15 rpm, 20 °C) in aqueous solution.

The pseudo-first and pseudo-second order models were employed to describe the kinetic data, using their non-linear forms, as reported in Table 6 and Figure 3. The AIC was again used as the estimator of the models’ relative quality. However, for a few materials, both kinetic models have very close AIC values (Δ ≤ 2), indicating that both models can fit the data properly [74,75]. For these cases, a joint analysis of these data with the isotherm model, and their parameters, was used to define the kinetic model for each adsorbent-adsorbate pair. In these situations, the pseudo-second order model was chosen because it was in agreement with the respective isotherm (Langmuir or Freundlich, with heterogeneity factor below 1), suggesting a chemisorption mechanism [19,76]. Moreover, the adsorption kinetics of these pollutants (or similar ones in the case of Rubi), when silica aerogels [33,36,40,77], ordered mesoporous silica [78] and amino-functionalized magnetic silica [79] are used, is usually described by the pseudo-second order model.

The kinetic results also reveal that the adsorption process is generally fast. This can be confirmed by the results presented in Table 6 and Figure 3.

For phenol and benzene adsorption, the equilibrium is practically achieved after 30 min; the pairs adsorbent-adsorbate with lower removal efficiencies (M_Phenol and MA_Benzene) showed higher values of *k*_2_, indicating a shorter time to reach the equilibrium, when compared with MA_Phenol and M_Benzene pairs. For the case of copper and lead, the process is fast at the beginning, but equilibrium is not reached, which can justify the significant differences between the experimental equilibrium and model uptake. In this case, the adsorption process continues slowly for several hours. This can be an indication that the amine groups became less available for adsorption after an initial uptake; hence, the process becomes slower. The same trend obtained for the VOCs was observed for the Pb removal, with MtA_Pb having a significantly lower value of *k*_2_ than Mt_Pb. In the case of the dyes, the adsorption process is also very fast, since after less than 30 min the equilibrium is already reached for both cases. The electrostatic interaction plays an important role in this kinetics, as the pollutants are attracted to the adsorbent due to the different surface charges, as already mentioned.

## 3. Materials and Methods

### 3.1. Materials

Methyltrimethoxysilane (MTMS, ≥98%, Sigma-Aldrich, St. Louis, MO, USA), methyltriethoxysilane (MTES, ≥ 99%, Sigma-Aldrich, Shanghai, China), tetramethylorthosilicate (TMOS, ≥98%, Sigma-Aldrich, Munich, Germany), tetraethylorthosilicate (TEOS, 98%, Sigma-Aldrich, Munich, Germany), (3-aminopropyl)trimethoxysilane (APTMS, ≥97%, Sigma-Aldrich, Munich, Germany) were used as silica precursors. Ethanol (EtOH, ≥99.8%, Fisher, Loughborough, England) and methanol (99.8%, VWR, Fontenay-sous-Bois, France) were used as solvents; anhydrous oxalic acid (≥99%, Sigma-Aldrich, Munich, Germany), ammonium hydroxide (25% NH_3_ in H_2_O, Sigma-Aldrich, Munich, Germany) and cetyltrimethylammonium bromide (CTAB, ≥99%, Acros, Mumbai, India) were used as catalysts and additives. Benzene (≥99%, Sigma-Aldrich, Dorset, England), phenol (≥99%, Sigma-Aldrich, Munich, Germany), copper(II) sulfate pentahydrate (>98%, Sigma-Aldrich, Munich, Germany), lead(II) nitrate (≥99.0%, Sigma-Aldrich, Dorset, England), Methylene Blue (VWR, Leuven, Belgium), Rubi Levafix CA reactive azo-dye (DyStar, Mem Martins, Portugal), sodium chloride (≥98%, Sigma-Aldrich, St. Louis, MO, USA), potassium hydroxide (pure, Sigma-Aldrich, Munich, Germany) and high purity water were used to prepare the pollutants solutions. The chemical structure of Rubi Levafix dye is not yet disclosed. All chemicals were used as received, unless stated otherwise.

### 3.2. Synthesis of Silica Aerogels

The adsorbents were synthesized through a sol-gel methodology following a one-step base catalyzed or a two-step acid-base catalyzed process, always conducted at 27 °C. The synthesis details for each adsorbent are given below.

#### 3.2.1. Synthesis of VOC Adsorbents

**Scheme 2:**
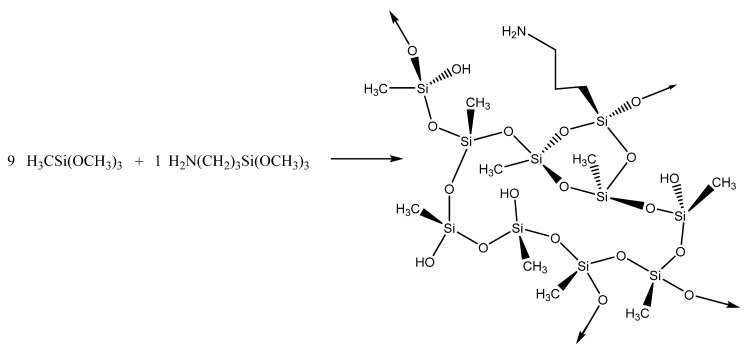
Illustration of amine-modified silica network of adsorbent MA.

The benzene and phenol aerogel adsorbents were synthesized using MTMS as precursor (adsorbent M), as reported in a previous work [54]. The influence of amine groups was assessed by adding APTMS as co-precursor (adsorbent MA), with MTMS:APTMS molar ratio of 90:10 [54]. The Si:solvent:acid water:basic water molar ratios were 1:12:4:4. MTMS was diluted in a mixture of ethanol/water (50/50 *v*/*v*) and the acid catalyst (oxalic acid, 0.01 M aqueous) and surfactant (CTAB, 1.25 g) were added. Then, APTMS was introduced into the system, for sample MA only (Scheme 2), followed by the basic catalyst (ammonium hydroxide, 1 M aqueous). Gelation occurred within 15 min for M samples and in 3 min for MA samples. The samples were aged for 7 days, washed afterwards eight times with ethanol at 60 °C, and finally dried at the same temperature for three days followed by 3 h at 100 °C.

#### 3.2.2. Synthesis of Heavy Metal Adsorbents

**Scheme 3:**
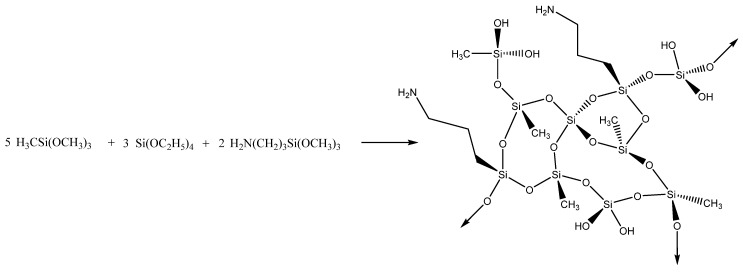
Illustration of amine-modified silica network of adsorbent MtA.

The adsorbents for heavy metals were synthesized similarly to a previous work [44]. MTES and TEOS (adsorbent Mt), in a 62.5:37.5 molar % respectively, or MTES, TEOS and APTMS (adsorbent MtA, Scheme 3), with 50:30:20 molar %, respectively, were diluted in methanol with the acid catalyst (oxalic acid, 0.1 M aqueous). The Si:solvent:acid water:basic water ratios were kept at 1:12:4:4. After sufficient hydrolysis, the basic catalyst (ammonium hydroxide, 1 M aqueous) was added. Gelation occurred within two hours for the first sample and ten minutes for the latter. The samples were aged for 6 days and dried in an oven at 60 °C for 3 days. For comparison purposes, the alcogels were also dried with supercritical CO_2_ [80], after being washed with hot ethanol, to obtain aerogels (adsorbents A_Mt and A_MtA).

#### 3.2.3. Synthesis of Dye Adsorbents

**Scheme 4:**
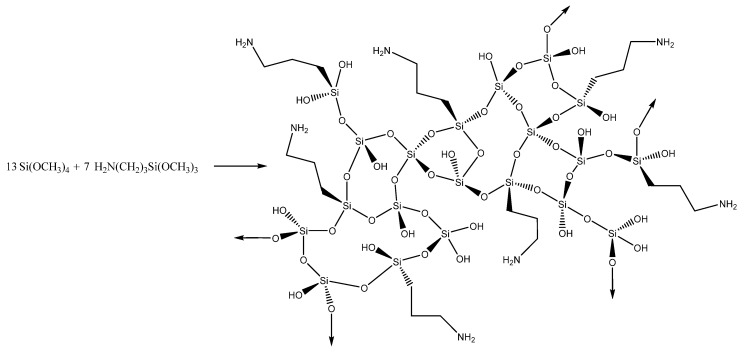
Illustration of amine-modified silica network of adsorbent TA.

For the adsorption of the reactive dye, TMOS aerogels (adsorbent T) were obtained, with Si:solvent:basic water molar ratio of 1:12:4. The effect of amine groups was studied by adding APTMS to TMOS (adsorbent TA, Scheme 4), with TMOS:APTMS = 65:35 molar % and the same molar ratios above mentioned. The co-precursors were mixed with methanol and basic catalyst (ammonium hydroxide, 1M aqueous), under stirring. Gelation occurred within minutes. The samples were aged for 5 days and dried in an oven during one day at 60 °C followed by 3 h at 100 °C. Aerogel adsorbents were also prepared (A_T and A_TA), by supercritically drying the alcogels using the same procedure as described in Section 3.2.2.

### 3.3. Characterization

Bulk density was calculated by measuring the mass and volume of portions of the sample. For samples that can be cut into regular forms, the volume was assessed by measuring their dimensions in the three axes; for irregular pieces, it was obtained by liquid displacement. Skeletal density was obtained using helium pycnometry on powdered samples (Accupyc 1330, Micrometrics). Porosity was calculated using bulk and skeletal densities, as mentioned elsewhere [54,81]. The Brunauer Emmett Teller (BET) specific surface area was obtained through nitrogen adsorption (ASAP 2000, Micrometrics), and the average pore size was calculated as described previously [54,81]. Hydrophobicity was assessed by contact angle measurement (OCA 20, Dataphysics) using the sessile drop technique and high-purity water. In samples that do not feature plane surfaces, the contact angle was determined on pellets of the compressed powdered sample.

The microstructures of the prepared samples were analyzed through scanning electron microscopy (SEM) using a Hitachi FEG-SEM SU70, after being coated with a conductive carbon thin layer, and Compact/VPCompact FESEM (Zeiss Merlin), after being coated with a thin gold layer.

The chemical composition was assessed by Fourier transform infrared (FTIR) spectra (FT/IR 4200, Jasco), which were obtained with KBr pellets of each sample, from 4000 to 400 cm^-1^ of wavenumber, 128 scans and 4 cm^−1^ of resolution.

In order to assess the incorporation of the amine groups into the silica structure, elemental analysis of previously grounded samples (EA 1108 CHNS-O, Fision Instruments), in terms of C, H and N elements, was performed.

To assess the surface charge of the dyes’ adsorbents, zeta potential technique was applied. After dispersing the samples (A_T and A_TA) in ultrapure water and sonicating for 30 min, zeta potential measurements were performed, at 25 °C, on a Malvern Instruments Zetasizer Nano-ZS. The zeta potentials were evaluated with Zetasizer 7.11 software.

Adsorbate concentrations in the adsorption tests were quantified with a UV-visible spectrophotometer (T70, PG Instruments) for VOC and dye pollutants, using specific wavelengths: 254, 272, 518 and 665 nm, for benzene, phenol, Rubi dye and methylene blue respectively; or with flame atomic absorption spectroscopy (Perkin Elmer 3000) for heavy metals.

### 3.4. Adsorption Experiments

The adsorbents were obtained from the prepared aerogels and xerogels after milling and sieving to obtain particles of 75–250 μm. The adsorbate solutions were prepared with high purity water. The Rubi Levafix CA dye adsorbate solution was prepared by mixing it with sodium chloride and potassium hydroxide at room temperature. In addition, this solution was heated up to 60 °C during 45 min and left to cool prior to its use. When necessary the pH of the solutions was adjusted to suitable values: 5 in the case of HMs [40,82,83].

The adsorption tests were conducted by placing the adsorbent in contact with the adsorbate solution in a test flask under shaking—Heidolph–REAX 20 shaker, at 16 rpm and 20 °C. The adsorbent concentration in the test flask was kept at 2 g/L. The used concentrations were selected to have similar values to real cases reported in the literature. In the equilibrium tests, the initial concentrations of the solutions varied between 10–200 mg/L for benzene and phenol [63,84]; 10–500 mg/L for copper and lead [24]; and 10–100 mg/L for MB and Rubi Levafix CA solutions [85].

All the solutions were shaken for 24 h to ensure equilibrium conditions. The kinetic experiments were conducted at different time intervals, from 2 min to 360 min. For benzene and phenol, the solutions have an initial concentration of 100 mg/L, for heavy metals this value was 200 mg/L [24], while for the dye the concentration was 50 mg/L.

After each test was concluded, the solutions were filtered, and their concentration determined by the techniques described in Section 3.3.

The equilibrium adsorption capacity, *q*_e_ (mg/L), is defined by Equation (1), where *C*_0_ (mg/L) and *C*_e_ (mg/L) are the initial and equilibrium adsorbate concentrations, respectively, *m* (g) is the mass of the adsorbent and *V* (L) the volume of the solution:

(1) $$q_{e}=\frac{V(C_{0}-C_{e})}{m}$$

The removal efficiency (*RE*) of the pollutants was calculated by Equation (2):

(2) $$RE(\%)=\frac{\left(C_{0}-C_{e}\right)}{C_{0}}\text{×}100$$

In order to understand the interaction between the adsorbent and adsorbate, two isotherm models were studied in this work, the Langmuir and Freundlich isotherm models. The models were fitted to the data using non-linear fitting algorithms. The Langmuir isotherm is described by Equation (3), where *q*_max_ is the monolayer adsorption capacity of the adsorbent (mg/g) and *K*_L_ is the Langmuir equilibrium constant (L/mg):

(3) $$q_{e}=\frac{q_{\max}K_{L}C_{e}}{1+K_{L}C_{e}}$$

The Freundlich isotherm is described by Equation (4). The parameters *K*_F_ and *n*_F_ refer to the Freundlich constant ((mg/g) (L/mg)^1/n^_F_) and to the heterogeneity factor, respectively:

(4) $$q_{e}=K_{F}C_{e}{}^{1/n_{F}}$$

The rate of the adsorption process was evaluated by fitting two empirical kinetic models to the data, namely the pseudo-first and pseudo-second order models, as described by Equations (5) and (6) (after integration with appropriate boundary conditions) [86,87,88]:

(5) $$q_{t}=q_{e}\left(1-e^{-tk_{1}}\right)$$

(6) $$q_{t}=\frac{q_{e}^{2}k_{2}t}{q_{e}k_{2}t+1}$$

where *k*_1_ (1/min) and *k*_2_ (g/(mg. min)) are the pseudo-first and pseudo-second order rate constants, respectively.

Akaike’s information criteria (AIC), Equation (7), was the chosen methodology for the evaluation of the best model [89]:

(7) $$\mathrm{AIC}=n\log\left(\frac{s^{2}}{n}\right)+2K$$

where *s*^2^ is the residual sum of squares, *n* is the number of experimental data points and *K* is the number of model parameters.

## 4. Conclusions

The possibility of changing the silica aerogels/xerogels characteristics allows the tailoring of these adsorbents to better remove different pollutants from wastewaters. The presence of amine groups has a significant impact in the materials properties. This chemical modification causes an increase in the adsorbents’ density, substantial reduction in their specific surface area and higher average pore size. Moreover, the amine moieties can change the surface charge as well as the hydrophilic character of the material. In this work, we proved that amine modification played a major role in the removal of different types of pollutants. The MTMS-based APD aerogels were able to effectively remove VOCs, achieving removals of up to 65% for benzene. In the case of phenol, removals of near 30% were obtained, with the amine presence duplicating the removal efficiency of this kind of adsorbent in high initial concentrations. In the case of dyes, the amine-modified aerogels were able to almost completely remove the industrial dye Rubi Levafix CA from the aqueous solutions, in a concentration up to 50 mg/L, leading to ~8-fold higher efficiency if compared with the A_T aerogel. For the heavy metals, the amine modification was also the key factor, with MtA materials showing removal efficiencies always above 70%. For most of materials, the adsorption was very fast, achieving the equilibrium in less than one hour. After this analysis, it is noteworthy the presence of amine groups as an important tool to the development of new adsorbents, by tuning their properties in order to enhance the adsorption of relevant pollutants. Therefore, this work clearly demonstrates the high potential of these materials to be used as alternative industrial sorbents due to their higher and fast removal efficiency towards different types of pollutants.

## Figures and Tables

**Table 1:** Chemical systems nomenclature, type of materials, silica precursors proportion and the adsorption capacity of the adsorbents for the studied pollutants.

				Adsorption Performance (mg/g)	
Adsorbent	Type of Material	Precursor system^(a)^	Initial concentration (mg/L)	Phenol	Benzene
M	Aerogel-like	100% MTMS	200	8.4 ± 0.8	51.0 ± 1.4
MA	Aerogel-like	90%MTMS/10%APTMS		19.1 ± 0.9	15.4 ± 1.6
				Copper	Lead
Mt	Xerogel	62.5%MTES/37.5%TEOS	500	14.8 ± 10.1	23.3 ± 1.5
A_Mt	Aerogel	62.5%MTES/37.5%TEOS		(b)	21.7 ± 0.6
MtA	Xerogel	50%MTES/30%TEOS/20%APTMS		124.2 ± 10.0	124.2 ± 2.5
A_MtA	Aerogel	50%MTES/30%TEOS/20%APTMS		115.6 ± 6.9	207.5 ± 2.1
				Rubi Levafix	Methylene Blue
T	Xerogel	100%TMOS	100	(b)	15.1 ± 0.3
A_T	Aerogel	100%TMOS		5.4 ± 0.6	12.2 ± 0.5
TA	Xerogel	65%TMOS/35%APTMS		37.6 ± 0.2	0.3 ± 0.1
A_TA	Aerogel	65%TMOS/35%APTMS		44.1 ± 0.1	(b)

(a) MTMS—methyltrimethoxysilane; APTMS—(3-aminopropyl)trimethoxysilane; MTES—methyltriethoxysilane; TEOS—tetraethylorthosilicate; TMOS—tetramethylorthosilicate (b) The adsorbent does not adsorb the pollutant.

**Table 2:** Physical and structural properties of the adsorbents.

Sample	Bulk Density/(kg/m^3^)	Skeletal Density/(kg/m^3^)	Porosity/(%)	*S*_BET_/(m^2^/g)	*D*_pore_^(a)^/(nm)	Contact Angle/(°)
M [54]	75.3 ± 5.1	1610 ± 75	95.3 ± 0.1	458.1 ± 2.2	110.5 ± 6.9	162.9 ± 3.9
MA [54]	80.9 ± 7.2	1479 ± 52	94.5 ± 0.3	72.2 ± 1.3	647.4 ± 46.1	164.4 ± 10.1
Mt	1068 ^(b)^	1400 ± 17	23.8	758.9 ± 15.5	1.2	94.7 ± 3.2
MtA	1410 ^(b)^	1459 ± 30	3.4	27.9 ± 0.1	3.5	(c)
A_T	70.4 ± 5.3	1793 ± 80	96.1 ± 0.1	817.2 ± 7.3	66.8 ± 4.5	(c)
A_TA	99.4 ± 4.4	1588 ± 50	93.7 ± 0.1	191.6 ± 3.6	197.0 ± 8.9	(c)

(a) Average pore diameter (*D*_pore_) was determined by 4*V*_P_/*S*_BET_. (b) Indicative value as it was determined by an immersion method. (c) The droplet is absorbed by the sample.

**Table 3:** Experimental and theoretical percentages of chemical elements in the samples.

Sample		wt% Si + O ^(a)^	wt% C	wt% H	wt% N
M	Experimental	74.60	20.30 ± 0.39	4.70 ± 0.04	0.41 ± 0.03
	Complete condensation	77.6	17.90	4.51	0.00
	Incomplete condensation 1OH	78.93	15.78	5.30	0.00
	Incomplete condensation 2OH	79.97	14.11	5.92	0.00
MA	Experimental	74.17	18.81 ± 0.35	5.05 ± 0.10	1.96 ± 0.02
	Complete condensation	72.92	20.18	4.94	1.96
	Incomplete condensation 1OH	74.70	17.92	5.64	1.74
	Incomplete condensation 2OH	76.12	16.11	6.20	1.57
Mt	Experimental	84.03	11.87 ± 0.17	3.52 ± 0.05	0.58 ± 0.03
	Complete condensation	85.43	11.64	2.93	0.00
	Incomplete condensation 1OH	85.84	10.22	3.94	0.00
	Incomplete condensation 2OH	86.17	9.10	4.73	0.00
MtA	Experimental	76.92	15.31 ± 1.12	4.47 ± 0.04	3.30 ± 0.06
	Complete condensation	74.01	17.95	4.24	3.81
	Incomplete condensation 1OH	75.62	15.99	5.00	3.39
	Incomplete condensation 2OH	76.92	14.42	5.61	3.06
A_T	Experimental	93.02	5.13 ± 0.07	1.68 ± 0.17	0.17 ± 0.08
	Complete condensation	100.00	0.00	0.00	0.00
	Incomplete condensation 1OH	98.54	0.00	1.46	0.00
	Incomplete condensation 2OH	97.42	0.00	2.58	0.00
A_TA	Experimental	73.16	17.40 ± 0.59	4.26 ± 0.09	5.18 ± 0.09
	Complete condensation	74.07	16.25	3.64	6.32
	Incomplete condensation 1OH	75.36	14.56	4.42	5.66
	Incomplete condensation 2OH	76.63	13.19	5.06	5.13

(a) The values indicated for Si + O are the differences between the sum of the other elements and 100%.

**Table 4:** Parameters of non-linear Langmuir and Freundlich isotherms.

	Langmuir Model				Freundlich Model			
	*q*_m_ (mg/g)	*K*_L_ (L/mg)	*R*_L_	AIC*^(a)^*	*1/n*_F_	*K*_F_ ((mg/g) (L/mg)^1/n^)	AIC*^(a)^*	Maximum *q*_e_ Experimental (mg/g)
M_Phenol	13 ± 4	0.02 ± 0.01	0.23–0.86	21	0.5 ± 0.2	0.8 ± 0.7	24	10.0 ± 0.9
MA_Phenol	41 ± 8	0.005 ± 0.002	0.50–0.95	14	0.70 ± 0.03	0.54 ± 0.09	7	19.1 ± 0.9
M_Benzene	(b)	(b)	(b)	--	0.7 ± 0.1	2.3 ± 1.0	--	51.0 ± 0.6
MA_Benzene	20 ± 3	0.03 ± 0.01	0.16–0.80	21	0.4 ± 0.1	1.9 ± 1.0	26	15.4 ± 0.2
Mt_Cu	(c)	(c)	(c)	(c)	(c)	(c)	(c)	14.8 ± 10.1
MtA_Cu	149 ± 11	0.018 ± 0.005	0.10–0.84	35	0.38 ± 0.01	15.0 ± 0.6	9	124.2 ± 10.0
Mt_Pb	25 ± 1	0.08 ± 0.03	0.07–0.80	30	0.23 ± 0.08	6.1 ± 2.7	39	24.0 ± 0.4
MtA_Pb	128 ± 4	0.11 ± 0.02	0.02–0.48	27	0.144 ± 0.001	55.9 ± 0.4	−11	124.2 ± 3.0
A_T_Rubi	12 ± 4	0.01 ± 0.01	0.50–0.91	8	0.7 ± 0.1	0.3 ± 0.2	9	5.6 ± 0.5
A_TA_Rubi	53 ± 3	0.4 ± 0.1	0.03–0.20	25	0.29 ± 0.02	21.5 ± 0.9	15	44.1 ± 0.1
A_T_Blue	13 ± 1	0.2 ± 0.1	0.04–0.32	29	0.22 ± 0.04	4.9 ± 0.7	24	12.7 ± 0.6
A_TA_Blue	(c)	(c)	(c)	(c)	(c)	(c)	(c)	0.2 ± 0.1

(a) AIC—Akaike’s Information Criteria; (b) the model does not fit well to the data; (c) the adsorption capacity is negligible or residual.

**Table 5:** Removal adsorption efficiencies of the materials for different pollutants against initial concentrations, *C*_0_, used in the equilibrium tests.

	Removal Efficiency (%)		
	*C*_0_ = 50 mg/L	*C*_0_ = 100 mg/L	*C*_0_ = 200 mg/L
M_Phenol	17.9 ± 1.8	20.3 ± 0.9	8.4 ± 1.7
MA_Phenol	28.4 ± 0.4	24.3 ± 0.8	19.1 ± 1.8
M_Benzene	61.6 ± 0.3	64.1 ± 1.8	51.0 ± 1.3
MA_Benzene	32.5 ± 0.4	30.9 ± 0.2	15.4 ± 1.2
Mt_Cu	(a)	(a)	(a)
MtA_Cu	94.1 ± 0.4	86.0 ± 0.5	70.2 ± 3.0
Mt_Pb	34.0 ± 2.0	21.3 ± 2.1	24.0 ± 0.4
MtA_Pb	100.0 ± 0.0	100.0 ± 0.0	88.2 ± 0.5
	*C*_0_ = 25 mg/L	*C*_0_ = 50 mg/L	*C*_0_ = 100 mg/L
A_T_Rubi	14.2 ± 2.1	14.3 ±1.7	10.9 ± 1.3
A_TA_Rubi	99.4 ± 0.1	97.6 ± 0.2	88.14 ± 0.2
A_T_Blue	62.8 ± 0.8	41.0 ± 0.1	24.5 ± 0.9
A_TA_Blue	(a)	(a)	(a)

(a) The removal efficiency is negligible.

**Table 6:** Kinetic model parameters for the studied pollutants on the silica-based xerogels and aerogels.

		Pseudo-First Order			Pseudo-Second Order			
Sample	*C*_0_ (mg/L)	*k*_1_ (1/min)	*q*_e_ (mg/g)	AIC (a)	*k*_2_ × 10^3^ (g/(mg.min))	*q*_e_ (mg/g)	AIC (a)	*q*_e (exp)_ (mg/g)
M_Phenol	100	0.25 ± 0.04	10.5 ± 0.2	−1	44.9 ± 11.4	10.8 ± 0.3	−2	10.0 ± 0.9
MA_Phenol	100	0.13 ± 0.02	16.1 ± 0.4	7	14.7 ± 3.2	16.9 ± 0.5	6	10.6 ± 0.6
M_Benzene	100	0.24 ± 0.03	37.1 ± 0.9	22	16.2 ± 7.2	38.1 ± 1.3	23	32.07 ± 0.03
MA_Benzene	100	0.35 ± 0.1	22.0 ± 0.5	13	38.7 ± 12.5	22.7 ± 0.4	6	15.5 ± 0.2
MtA_Cu	200	0.03 ± 0.01	17.4 ± 1.5	31	1.6 ± 0.5	20.6 ± 1.6	27	70.2 ± 3.0
Mt_Pb	200	0.32 ± 0.03	23.7 ± 0.2	17	37.8 ± 5.8	24.2 ± 0.3	18	24.0 ± 0.4
MtA_Pb	200	0.02 ± 0.01	26.0 ± 2.4	13	0.62 ± 0.2	33.0 ± 3.2	7	88.2 ± 0.5
A_TA_Rubi	50	0.19 ± 0.04	23.7 ± 0.7	18	12.7 ± 1.6	25.5 ± 0.4	8	24.40 ± 0.05
A_T_Blue	50	0.30 ± 0.05	10.7 ± 0.2	19	58.9 ± 6.0	11.3 ± 0.1	8	10.25 ± 0.04

(a) AIC—Akaike’s Information Criteria.

## Supplementary Materials

The following data are available online at https://www.mdpi.com/1420-3049/24/20/3701/s1, Figure S1: Comparative N_2_ sorption isotherms of the adsorbents with and without amine modification for (a) volatile organic compounds, (b) heavy metals and (c) dyes. Figure S2: FTIR spectra of adsorbent materials, Figure S3: FTIR spectrum of Rubi Levafix CA.
