# Supplementary material for: Amine Modification of Silica Aerogels/Xerogels for Removal of Relevant Environmental Pollutants
Source: Molecules. 2019 Oct 15;24(20):3701. doi: 10.3390/molecules24203701 (PMC6833102; doi:10.3390/molecules24203701)
Supplement: Supplementary file 1 [file molecules-24-03701-s001.pdf]

molecules

Article

Amine modification of silica aerogels/xerogels for removal of relevant environmental pollutants

Alyne Lamy-Mendes [1,2], Rafael B. Torres [1], João P. Vareda [1], David Lopes [1], Marco Ferreira [1], Vanessa Valente [1], Ana V. Girão [2], Artur J. M. Valente [3] and Luísa Durães [1,*]

[1] CIEPQPF, Department of Chemical Engineering, University of Coimbra, 3030-790 Coimbra, Portugal
[2] CICECO - Aveiro Institute of Materials, Department of Materials and Ceramic Engineering, University of Aveiro, 3810-193 Aveiro, Portugal
[3] CQC, Department of Chemistry, University of Coimbra, 3004-535 Coimbra, Portugal
* Corresponding author: luisa@eq.uc.pt (L. Durães).

Supplementary Material

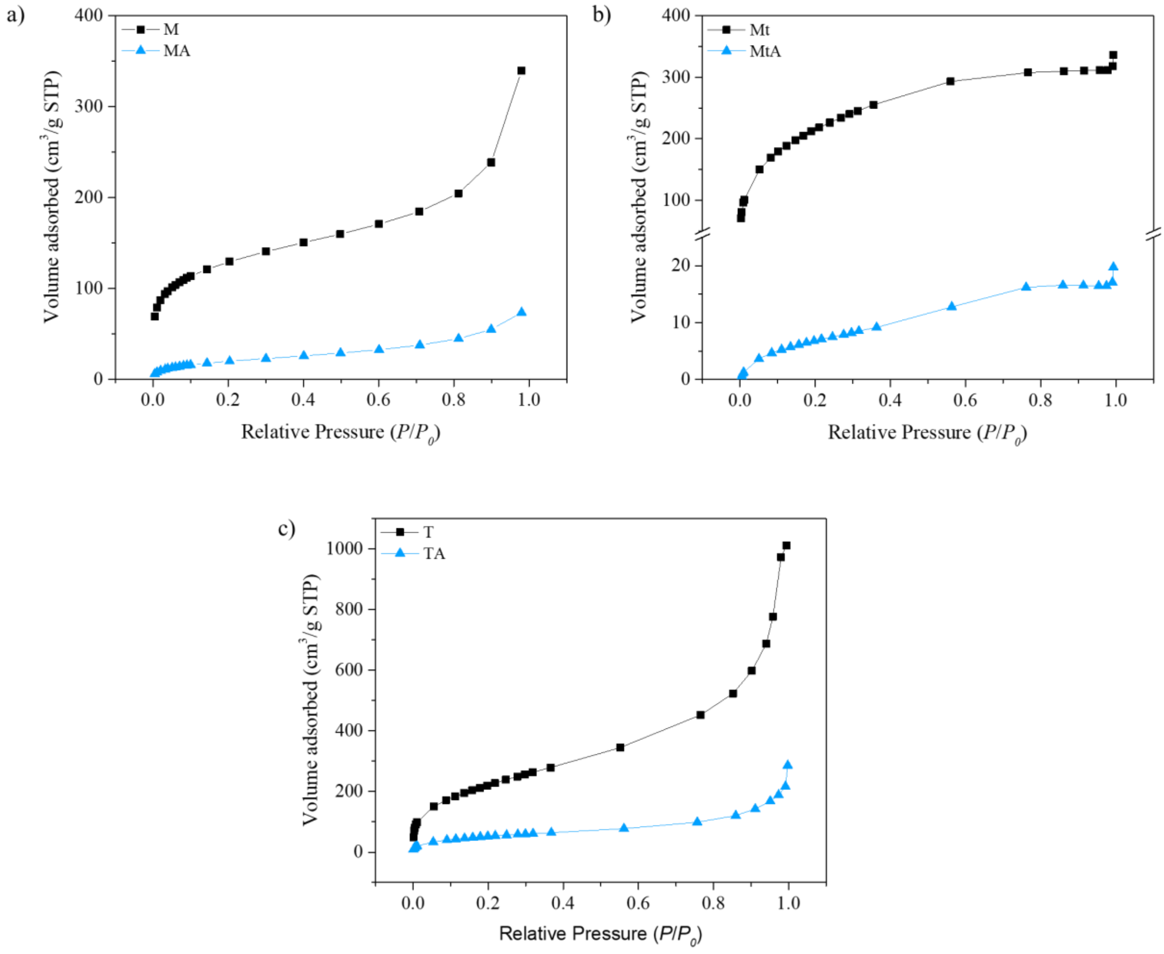

Figure 1. Comparative N_2 sorption isotherms of the adsorbents with and without amine modification for (a) volatile organic compounds, (b) heavy metals and (c) dyes.

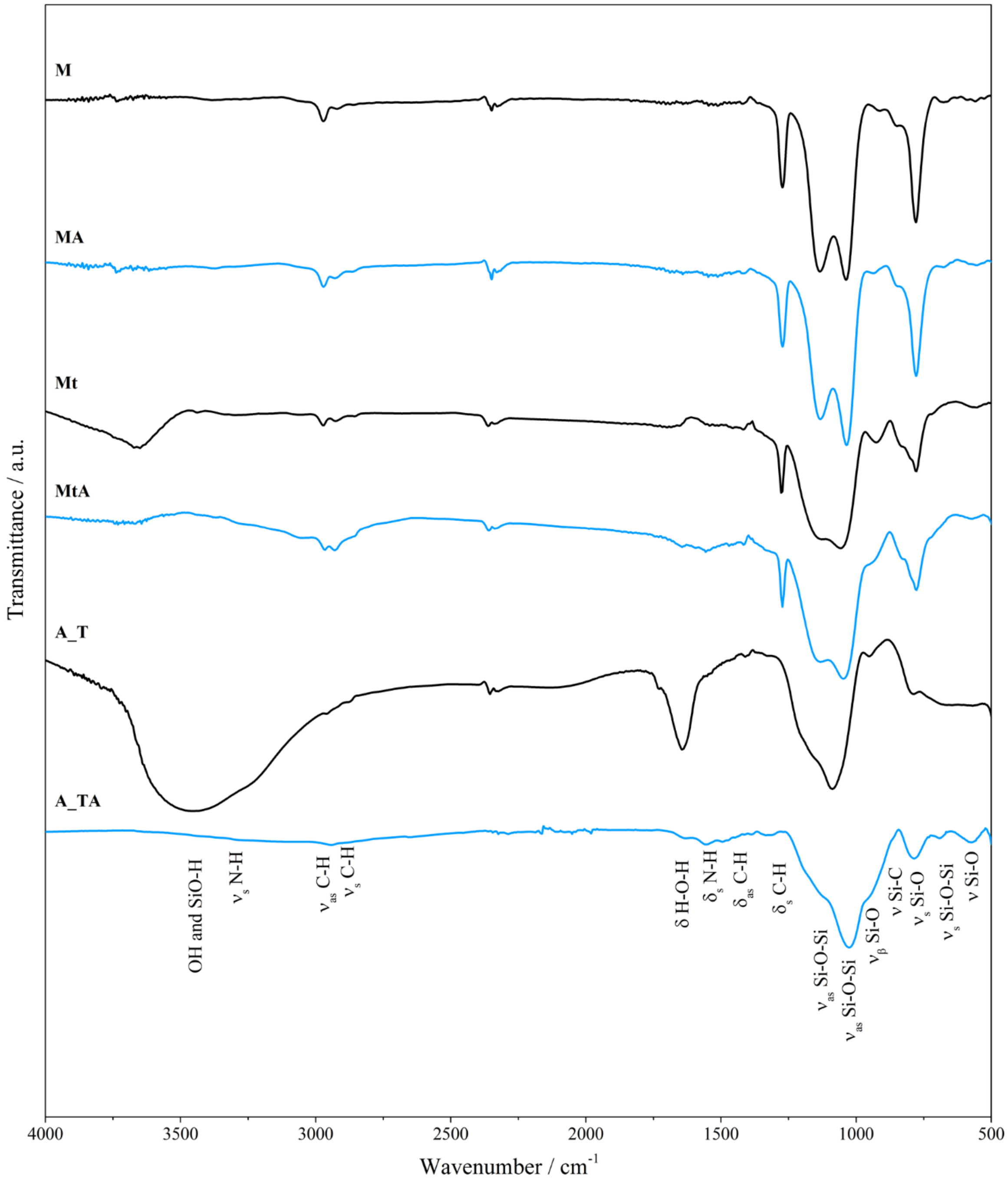

Figure 2. FTIR spectra of adsorbent materials (υ—stretching vibration, υ_s—symmetric stretching vibration; υ_{as}—asymmetric stretching vibration; υ_β—in-plane stretching vibration; δ—bending vibration; δ_s—symmetric deformation vibration (bending); δ_{as}—asymmetric deformation vibration (bending)).

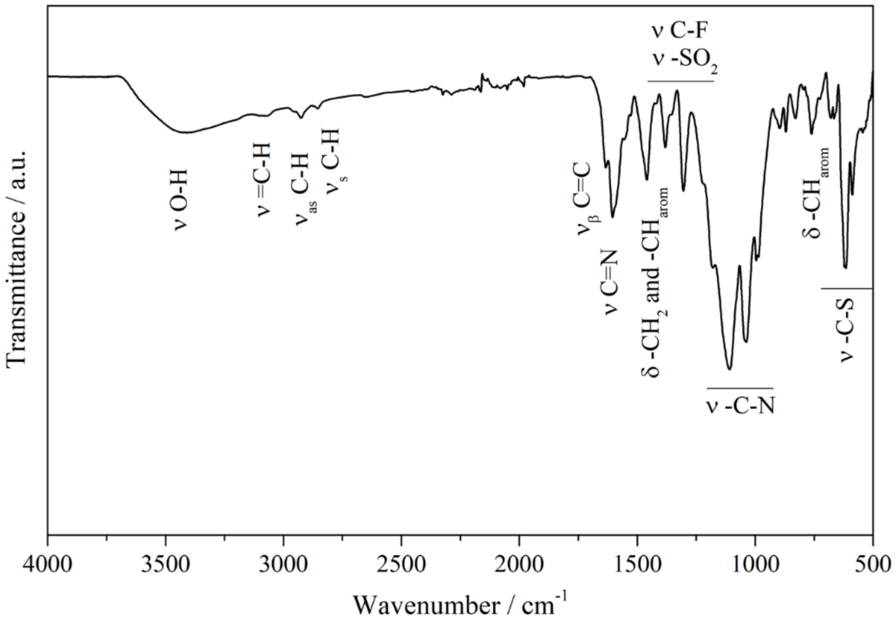

Figure 3. FTIR spectrum of *Rubi Levafix CA* (υ—stretching vibration, $υ_s$—symmetric stretching vibration; $υ_{as}$—asymmetric stretching vibration; $υ_β$—in-plane stretching vibration; δ—bending vibration; $δ_s$—symmetric deformation vibration (bending); $δ_{as}$—asymmetric deformation vibration (bending)).
